# Multiple physical aspects during the flow and heat transfer analysis of Carreau fluid with nanoparticles

**DOI:** 10.1038/s41598-018-35462-9

**Published:** 2018-11-27

**Authors:** Hashim, Abdul Hafeez, Ali Saleh Alshomrani, Masood Khan

**Affiliations:** 1 Department of Mathematics, Quaid-i-Azam University, Islamabad, 44000 Pakistan; 2 NAAM Research Group, Department of Mathematics, Faculty of Science, King Abdul-Aziz University, Jeddah, 21589 Saudi Arabia

## Abstract

The current work is concerned with the two-dimensional boundary layer flow of a non-Newtonian fluid in the presence of nanoparticles. The heat and mass transfer mechanism for Carreau nanofluid flow due to a radially stretching/shrinking sheet is further investigated in this article. The governing physical situation is modelled in the form of partial differential equations and are simplified to a system of non-linear ordinary differential equations by employing dimensionless variables. Numerical simulations for non-dimensional velocity, temperature and concentration fields has been performed with the assistance of built-in Matlab solver bvp4c routine. One significant computational outcome of this study is the existence of multiple numerical solutions for the flow fields. The impacts of various developing parameters, for instance, Weissenberg number, power-law index, shrinking parameter, suction parameter, Prandtl number, Schmidt number, Brownian motion and thermophoresis parameter on the velocity, temperature and nanoparticles concentration are visualized through tables and graphical experiment. The numerical results demonstrate that the rates of heat and mass transfer are raised by higher Weissenberg number for first solution and an inverse is seen for second solution. Moreover, an increasing trend is seen in nanofluids temperature for both solutions with greater values of thermophoresis parameter. In addition, the numerical results obtained by the applied technique are validated with existing literature and found to be in an excellent agreement.

## Introduction

In the field of fluid dynamics, the study of nanofluids has attracted the attention of many investigators because of many important technological processes and applications. In recent few decades, an attractive technique for heat transfer improvement in industrial systems is the usage of nanoparticles in the base fluids. Therefore, nanofluids are assumed to be a mixture of base fluid and nanoparticles (1–100 nm) which are uniformly dispersed in a base fluid. These disseminated nanoparticles, generally a metal or metal oxide enormously improve the thermal conductivity of the nanofluid, increases convection and conduction coefficients, permitting for more heat transfer. The situation transmuted when Choi and Eastman^1^ in Argonne National Laboratory revisited this field with their nanoscale metallic particle and carbon nanotube suspensions Eastman *et al*.^2^. Choi and Eastman have endeavoured to suspend different metal and metal oxides nanoparticles in numerous distinct fluids and their outcomes are encouraging, moreover, many stuff remain elusive about these suspensions of nano-organized materials, which have been named nanofluids by Choi and Eastman. Nanofluid is basically the mixture of base fluid and nanoparticle. It substantially contributes in nanotechnology in the invention of functional devices, material and system by controlling the nanoscale level. Firstly, the concept of nanoparticles was given by Choi and Eastman^1^, many research workers are adopted to formulate the heat and transfer characteristics of nanofluid flows. Later, in 2006, Buongiorno^3^ presented a complete and thorough study about the heat transport in nanofluids. In his work he found an amazing elevate in the thermal conductivity of nanofluids. He proposed a model which disregards the restrictions of dispersion and homogeneous models. He exhibited the seven slip mechanisms that produce a parallel velocity between the nanoparticles and base fluid. These incorporate inertia, Magnus effect, Brownian diffusion, thermophoresis, fluid drainage and gravity, etc. He inferred that the thermophoresis and Brownian diffusion in nanofluids are two essential slip mechanisms. From these characteristics, many researchers have been worked the investigations on nanofluids. The investigation on boundary layer flow of a nanofluid over a stretching surface was studied by Khan and Pop^4^. This was the first attempt to study on boundary layer flow over a stretching surface via the use of a model in which thermophoresis and Brownian motion effects have been taken into consideration. A numerical and analytical study for the axisymmetric flow of nanofluid was reported by Mustafa *et al*.^5^. They see that increment in Schmidt number causes to a thinner nanoparticle boundary layer. Moreover, Hashim *et al*.^6^ numerically investigated the analysis of heat and mass transfer in the Carreau fluid model using Runge-Kutta technique in MATLAB. Ellahi *et al*.^7^ studied the impact of particle shape on Marangoni convection boundary layer flow of nanofluid. They enforced the nanoparticles mass flux and convective boundary conditions in this study. Sheikholeslami^8^ presented the effect of variable magnetic field on the flow of *Fe*_3_*O*_4_-*H*_2_*O* nanofluid in a cavity with circular hot cylinder. Innovative numerical method, namely CVFEM is selected to perform the numerical computations. Recently, the transport of nanofluids are investigated by many researchers, see ^9–12^.

It is prominent fact that studies about boundary layer flow on stretching/shrinking sheet have gained a great importance because of its ever-incrementing to do with industry applications in some technology-based cognitive process. Many cases are trans actioned with stretching/shrinking surface appearing in manufacturing of rubber and plastic sheet, polymer-industries, spinning of fibres etc. Scientists are fascinated to develop different methods acting to increment their heat transfer exhibition. The boundary layer flow over a stretching surface was firstly studied by Crane^13^. Later in 2010, Khan and Pop^4^ have disciplined the boundary layer rate of flow over a linear stretching sheet. Moreover, Wang^14^ was discussed the study for unsteady film solution on the boundary layer flow over a shrinking surface. After that, Rana *et al*.^15^ put into use finite element model for nonlinear stretching sheet to discuss the behaviour of flow and heat transfer. Specifically, flow over a stretching sheet with quadratic^16^, exponential^17^, nonlinear^18^ and oscillatory^19^ were discussed by different authors. For the case of exponentially stretching sheet, the skin friction at the wall detailed by Elbashbeshy^17^ is higher than that processed by Vajravelu^18^ for the nonlinear stretching sheet even with *u*_*w*_ = *cx*^5^. Then again, the skin friction at the wall has oscillatory conduct in the position of oscillatory stretching sheet as visualized by Abbas *et al*.^19^. The classical problem of axisymmetric flow because of radially stretching plat was discussed via Ariel^20^. Later, Sajid *et al*.^21^ who discussed about series solution for axisymmetric flow over a nonlinear stretching sheet. Similarly, Khan and Shehzad^22^ are performed the exact solution for steady axisymmetric flow due to nonlinear stretching sheet.

The notable investigation within the sight of the dual solutions for flow and heat transfer characteristics have been presented by several researchers. In this regard, Lio^23^ investigated the problem of boundary layer flows caused by a stretching surface. He employed the analytic method known as homotopy analysis technique to get the two branches of solutions. After that, Fang^24^ numerically studied the flow and heat transfer features for Newtonian fluid past a stretching sheet. He utilized the Runge-Kutta numerical integration scheme to get the dual solutions for flow fields. Dual solutions for stagnation-point flow in the presence of chemical reaction past a stretching/shrinking cylinder has been deliberated by Najib *et al*.^25^. Additionally, the flow and heat transfer characteristics in the presence of nanoparticles over a nonlinearly stretching/shrinking sheet has been examined by Zaimi *et al*.^26^. They observed that multiple solutions exist for a specific range of shrinking parameter. Recently, Naganthran^27^ bestowed a numerical review for the unsteady flow of third grade fluid past a stretching/shrinking sheet. The multiple branches of solution have been derived using bvp4c routine in MATLAB. Recently, Khan *et al*.^28^ also carried out a numerical simulation for slip-flow and heat transfer features of nanofluid past a permeable shrinking sheet in the presence of non-linear thermal radiation.

According to the literature review, a limited amount of research has been linked to evaluating flow and heat transfer characteristics for non-Newtonian fluids due to a radially shrinking sheet in the presence of nanoparticles. However, to the best knowledge of the authors, only few researchers have ever attempted to obtain the multiple solutions for the flow of a non-Newtonian Carreau nanofluids caused by a radially shrinking surface. Additionally, keeping in mind the engineering applications of nanofluids and flow caused by a radially shrinking surface, the main objectives and novelty of this article is:

i. To investigate the flow of Carreau nanofluids by employing Buongiorno’s model of nanfluid.
ii. The mathematical formulation is presented in the company of Brownian motion and thermophoresis.
iii. The flow analysis is examined in the neighbourhood of stagnation-point.
iv. The impacts of uniform suction are addressed in view of its physical features.
v. For numerical treatment, the use of a MATLAB routine bvp4c based on finite difference scheme to acquire the multiple solutions for current governing problem.
vi. The set of critical values obtained for different values of controlling parameter are explored graphically.

However, the thermo-physical aspects during the flow of non-Newtonian fluids generated by a stretching surface using nanofluid has various applications in manufacturing processes where the raw material passes through the die for the extrusion in a liquefied state under high temperature with densities gradient. Moreover, the phenomenon of stretching surface into a cooling medium is a mathematical tool for the process of heat treatment in the fields of engineering technology noticed in mechanical, civil, architectural engineering. After modelling the problem, the solution of nonlinear differential equations governing the flow problem has been carried out.

## Mathematical Model

**Figure 1:**
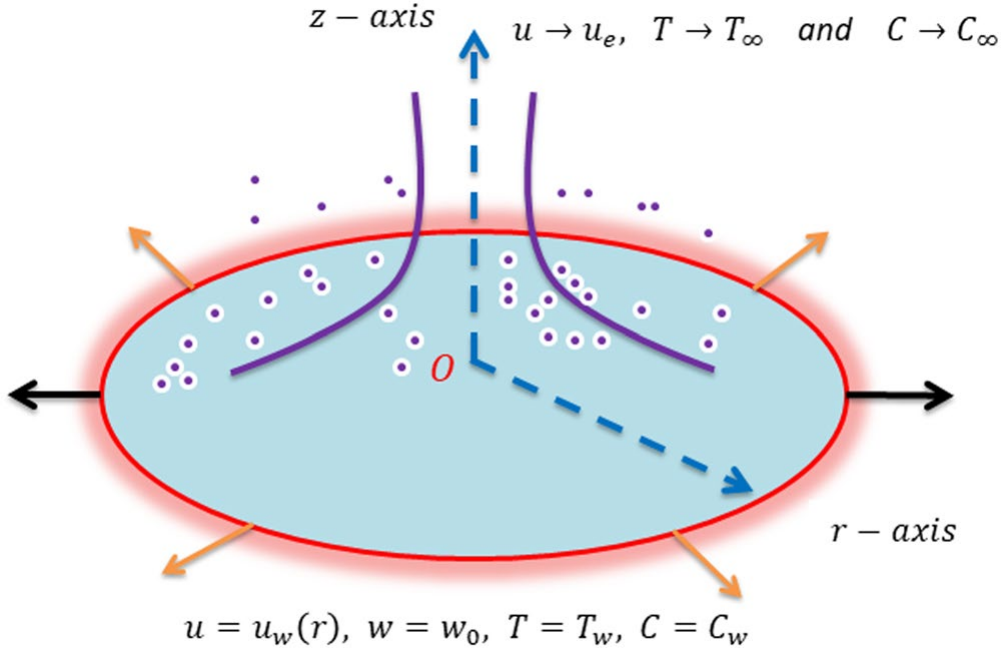
A Schematic of the physical problem and coordinates configuration.

Steady incompressible two-dimensional flow of a non-Newtonian Carreau nanofluid driven by a radially stretching/shrinking sheet is considered. Further, in this study heat and mass transfer in the presence of Brownian motion and thermophoresis are investigated. A locally orthogonal set of coordinates (*r*, *z*) is chosen in such a way that the origin *O* is kept in the plane of stretching/shrinking sheet. The velocity of the stretching/shrinking sheet is denoted as *u*_*w*_ = *ar*^*m*^ in which *a* and *m* are constants. The geometry of the physical problem is shown in Fig. 1. The sheet is kept at a constant temperature *T*_*w*_. Here, *T*_∞_ and *C*_∞_ are the ambient temperature and nanoparticle concentration, respectively. Moreover, the free stream velocity is *u*_*e*_(*r*) = *br*^*m*^ for which *b* is a constant.

The differential equations that model this problem consist of four categories of conservation of mass, momentum, energy and nanoparticle concentration equations, which are expressed as^3,4^:

1 $$\frac{{\boldsymbol{\partial }}u}{{\boldsymbol{\partial }}r}+\frac{u}{r}+\frac{{\boldsymbol{\partial }}w}{{\boldsymbol{\partial }}z}=0,$$

2 $$u\frac{{\boldsymbol{\partial }}u}{{\boldsymbol{\partial }}r}+w\frac{{\boldsymbol{\partial }}u}{{\boldsymbol{\partial }}z}=\nu \frac{{{\rm{\partial }}}^{2}u}{{\rm{\partial }}{z}^{2}}{[1+{{\rm{\Gamma }}}^{2}{(\frac{{\rm{\partial }}u}{{\rm{\partial }}z})}^{2}]}^{\frac{n-1}{2}}+\nu (n-1){{\rm{\Gamma }}}^{2}{(\frac{{\rm{\partial }}u}{{\rm{\partial }}z})}^{2}\frac{{{\rm{\partial }}}^{2}u}{{\rm{\partial }}{z}^{2}}{[1+{{\rm{\Gamma }}}^{2}{(\frac{{\rm{\partial }}u}{{\rm{\partial }}z})}^{2}]}^{\frac{n-3}{2}}+{u}_{e}\frac{d{u}_{e}}{dr},\,$$

3 $$(u\frac{{\rm{\partial }}T}{{\rm{\partial }}r}+w\frac{{\rm{\partial }}T}{{\rm{\partial }}z})=\alpha \frac{{{\rm{\partial }}}^{2}T}{{\rm{\partial }}{z}^{2}}+\tau [{D}_{B}(\frac{{\rm{\partial }}C}{{\rm{\partial }}z})\,(\frac{{\rm{\partial }}T}{{\rm{\partial }}z})+\frac{{D}_{T}}{{T}_{{\rm{\infty }}}}{(\frac{{\rm{\partial }}T}{{\rm{\partial }}z})}^{2}],$$

4 $$u\frac{\partial C}{\partial r}+w\frac{\partial C}{\partial z}={D}_{B}(\frac{{\partial }^{2}C}{\partial {z}^{2}})+\frac{{D}_{T}}{{T}_{\infty }}(\frac{{\partial }^{2}T}{\partial {z}^{2}}),$$

The corresponding boundary conditions for the stretching/shrinking sheet is

5 $$u={u}_{w}=a{r}^{m},\,w={w}_{0},\,T={T}_{w},\,C={C}_{w}\,{\rm{a}}{\rm{t}}\,z=0,$$

6 $$u={u}_{e}=b{r}^{m},\,T\to {T}_{\infty },\,C\to {C}_{\infty }\,{\rm{as}}\,z\to \infty .$$

Here, *u* and *w* denotes the velocity components along *r*− and *z*− directions, respectively, *ν*, *α*, *D*_*B*_, *D*_*T*_, *C*, *T* are the kinematic viscosity, thermal diffusivity, Brownian motion coefficient, thermophoresis diffusion coefficient, nanoparticles concentration, fluid temperature. Furthermore, *τ* refers for the ratio of nanoparticle heat capacity and nanofluid heat capacity, *n* and Γ denotes the power-law index and the material parameter also known as relaxation time. It is important to note that the Newtonian case is achieved for *n* = 1 or Γ = 0.

The non-dimensional variables for the governing Eqs (1–4) with boundary conditions (5) and (6) are written as follows:

7 $$\eta =\frac{z}{r}\,{{\rm{R}}{\rm{e}}}^{1/2},\,\psi =-\,{r}^{2}{u}_{e}(r){{\rm{R}}{\rm{e}}}^{-1/2}f,\,\theta (\eta )=\frac{T-{T}_{{\rm{\infty }}}}{{T}_{w}-{T}_{{\rm{\infty }}}},\,\phi (\eta )=\frac{C-{C}_{{\rm{\infty }}}}{{C}_{{\rm{\infty }}}},\,{\rm{R}}{\rm{e}}=\frac{r{u}_{e}(r)}{\nu }.$$

The velocity components in the perspective of stream function are takes after as:

8 $$u={u}_{e}f^{\prime} (\eta ),\,w=-\,{u}_{e}\,{{\rm{Re}}}^{-1/2}(\frac{m+3}{2}f(\eta )+\frac{m-1}{2}\eta f^{\prime} (\eta )).$$

Employing Eqs (7 and 8) into governing Eqs (2–4), we get:

9 $$(1+nW{e}^{2}{f^{\prime\prime} }^{2}){(1+W{e}^{2}{f^{\prime\prime} }^{2})}^{\frac{n-3}{2}}f\prime\prime\prime +(\frac{m+3}{2})\,ff^{\prime\prime} -m{f^{\prime} }^{2}+1=0,$$

10 $$\frac{1}{{\rm{\Pr }}}\theta ^{\prime\prime} +(\frac{m+3}{2})\,f\theta ^{\prime} +Nb\theta ^{\prime} \varphi ^{\prime} +Nt{(\theta ^{\prime} )}^{2}=0.$$

11 $$\varphi ^{\prime\prime} +\frac{m+3}{2}Scf\varphi ^{\prime} +\frac{Nt}{Nb}\theta ^{\prime\prime} =0.$$

The transformed boundary conditions are:

12 $$f=s,\,f^{\prime} =\chi ,\,\theta =1,\,\varphi (0)=1,\,{\rm{at}}\,\eta =0,$$

13 $$f^{\prime} =1,\,\theta =0,\,\varphi =0,\,{\rm{at}}\,\eta \to \infty ,$$

where, primes represent the differentiation with respect to *η*.

The different flow parameters appearing in Eqs (9–13) are characterized by:

$We={(\frac{{b}^{3}{{\rm{\Gamma }}}^{2}{r}^{3m-1}}{\nu })}^{1/2}$ is the local Weissenberg number, $s=\frac{{w}_{0}}{-\,2}{(\frac{\nu {u}_{e}}{r})}^{-1/2}$ the suction parameter, ${\rm{\Pr }}=\frac{{(\rho c)}_{f}v}{k}$ the Prandtl number, $\chi =\frac{a}{b}$ the stretching/shrinking parameter, $Nb=\frac{{(\rho c)}_{p}{D}_{B}{C}_{\infty }}{{(\rho c)}_{f}v}$ the Brownian motion and $Nt=\frac{{(\rho c)}_{p}{D}_{T}({T}_{w}-{T}_{\infty })}{{(\rho c)}_{f}v{T}_{\infty }}$ thermophoresis parameters, $Sc=\frac{v}{{D}_{B}}$ the Schmidt number.

The parameters of physical and engineering importance are the skin friction, local Nusselt number and local Sherwood number. In mathematical form, these are expressed as:

**Skin-friction coefficient:** The skin friction coefficient (wall shear stress) *C*_*f*_ is given as:

14 $${C}_{f}={\frac{\mu }{{\rho }_{f}{u}_{e}^{2}}\frac{{\boldsymbol{\partial }}u}{{\boldsymbol{\partial }}z}{[1+{{\rm{\Gamma }}}^{2}{(\frac{{\rm{\partial }}u}{{\rm{\partial }}z})}^{2}]}^{\frac{n-1}{2}}|}_{z=0}.$$

Upon using Eqs (7 and 8), the dimensionless form of skin friction becomes

15 $${{\rm{Re}}}^{1/2}{C}_{f}=f^{\prime\prime} (0){[1+W{e}^{2}{(f^{\prime\prime} (0))}^{2}]}^{\frac{n-1}{2}}.$$

**Local Nusselt number:** The local Nusselt number (rate of heat transfer) *Nu* is given as:

16 $$Nu=\frac{{-r\frac{{\boldsymbol{\partial }}T}{{\boldsymbol{\partial }}z}|}_{z=0}}{({T}_{w}-{T}_{\infty })}.$$

In view of non-dimensional variables (7), the reduced form of local Nusselt number is

17 $${{\rm{Re}}}^{-1/2}Nu=-\,\theta ^{\prime} (0).$$

**Local-Sherwood number:** The local Sherwood number (rate of mass transfer) *Sh* is written as:

18 $$Sh=\frac{{r\frac{{\boldsymbol{\partial }}C}{{\boldsymbol{\partial }}z}|}_{z=0}}{({C}_{w}-{C}_{\infty })}.$$

Making use of Eq (7), local Sherwood number  reduces to

19 $${{\rm{Re}}}^{-1/2}Sh=-\,\varphi ^{\prime} (0).$$

### Numerical approach

The set of governing Eqs (9–11) are non-linear in nature and their exact solutions are not feasible. Therefore, the transformed set of ordinary differential Eqs (9–11) alongside the boundary conditions (12) and (13) are numerically integrated via the boundary value problem solver bvp4c in MATLAB. The main theme of this package utilized the finite difference technique. In this method, the system of partially coupled differential equations is altered to a set of first order ordinary differential equations. To do this, let us define the new variables

20 $$f={y}_{1},\,f^{\prime} ={y}_{2},\,f^{\prime\prime} ={y}_{3},\,\theta ={y}_{4},\,\theta ^{\prime} ={y}_{5},\,\varphi ={y}_{6},\,\varphi ^{\prime} ={y}_{7}.$$

After transformed into first order equation becomes

21 $${y{\rm{^{\prime} }}}_{1}={y}_{2},\,{y{\rm{^{\prime} }}}_{2}={y}_{3},\,{y{\rm{^{\prime} }}}_{3}=\frac{m{y}_{2}-(\frac{m+3}{2}){y}_{1}{y}_{3}-1}{(1+nW{e}^{2}{y}_{3}^{2}){(1+W{e}^{2}{y}_{3}^{2})}^{\frac{n-3}{2}}},$$

22 $${y^{\prime} }_{4}={y}_{5},\,{y^{\prime} }_{5}=-\,{\rm{\Pr }}({y}_{1}{y}_{5}+Nb{y}_{5}{y}_{7}+Nt{y}_{5}^{2}),$$

23 $${y^{\prime} }_{6}={y}_{7},\,{y^{\prime} }_{7}=-\,(Le{y}_{1}{y}_{7}+\frac{Nt}{Nb}{y^{\prime} }_{5}),$$

with the associated initial conditions as

24 $${y}_{1}(0)=s,\,{y}_{2}(0)=\chi ,\,{y}_{4}(0)=1,\,{y}_{7}(0)=1,$$

25 $${y}_{3}(\infty )=1,\,{y}_{4}(\infty )=0,\,{y}_{6}(\infty )=0.$$

The above system of seven first order differential Eqs (21–23) with initial conditions (24) and (25) can be solved with the bvp4c function in MATLAB. The maximum residual error is considered here is 10^−5^. In this scheme, the dual solutions are collected by adjusting different initial guesses for *y*_3_(∞) and *y*_4_(∞) i.e., *f*″(0) and *θ*′(0) according to the different physical parameters. Moreover, the far field conditions (25) has to be satisfied asymptotically by all the profiles. In this analysis, the boundary conditions (25) are implemented for some finite value of similarity variable *η* denoted by ${\eta }_{\max }$. We did our computations for *η*_max_ = 7, 10 and 15 to achieve asymptotic behaviour of parameters on velocity, temperature and concentration profiles.

### Validation of numerical scheme

To establish the accuracy of our computational scheme, a comparison is made for the numeric data of local skin friction coefficient and local Nusselt number in limiting cases with that of Wang and Ng^29^, Rosca *et al*.^30^, Soid *et al*.^31^ and Wang *et al*.^32^. These comparisons are depicted in Tables 1 and 2. These tables demonstrate an outstanding agreement between the present numerical results and the existing works. This gives us reliance of our numerical outcomes.

**Table 1:** A comparison of the skin friction *Re*^1/2^*C*_*f*_  and Nusselt number *Re*^−1/2^*Nu*, when *s* = 0.0 = *We* = *χ* and *n* = 1.0.

	*Re* ^1/2^ *C*_*f*_	*Re* ^−1/2^ *Nu*	
		Pr = 0.7	Pr = 7
Wang and Ng^29^	1.31194	−0.6654	−1.5458
Rosca *et al*.^30^	1.311937	—	—
Soid *et al*.^31^	1.311938	—	—
Present Study	1.3119374	−0.66537722	−1.5457923

**Table 2:** A comparison of the skin friction *Re*^1/2^*C*_*f*_  for various values of shrinking parameter *χ*, when *s* = 0.0 = *We* and *n* = 1.0.

*χ*	−0.25	−0.5	−0.75	−0.95
Wang *et al*.^32^	1.45664	1.49001	1.35284	0.94690
Present Study	1.4566387	1.4900101	1.3528388	0.94690336

## Results and Discussion

**Figure 2:**
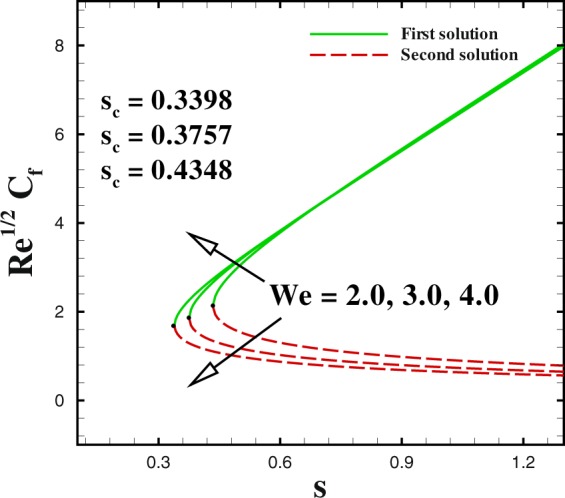
Impact of *We* on skin friction.

**Figure 3:**
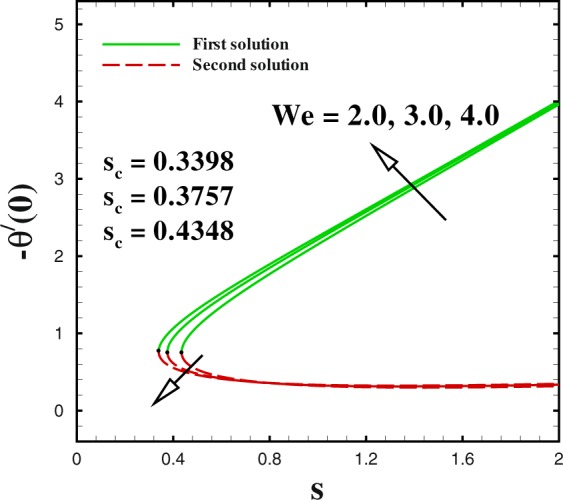
Impact of *We* on Nusselt number.

**Figure 4:**
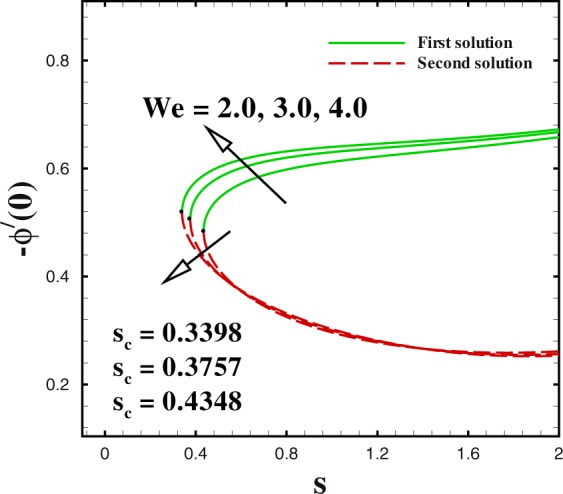
Impact of *We* on Sherwood number.

**Figure 5:**
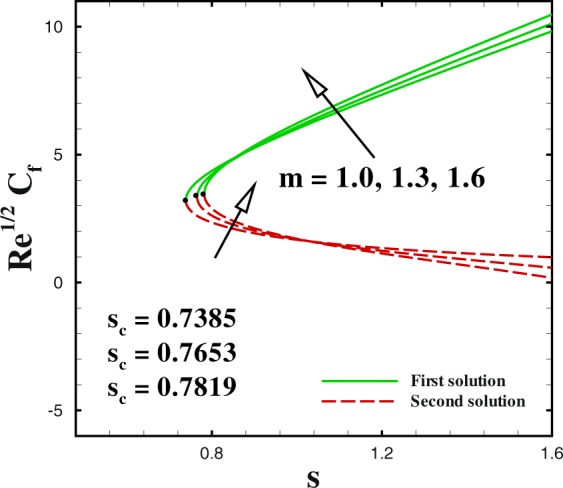
Impact of *m* on skin friction.

**Figure 6:**
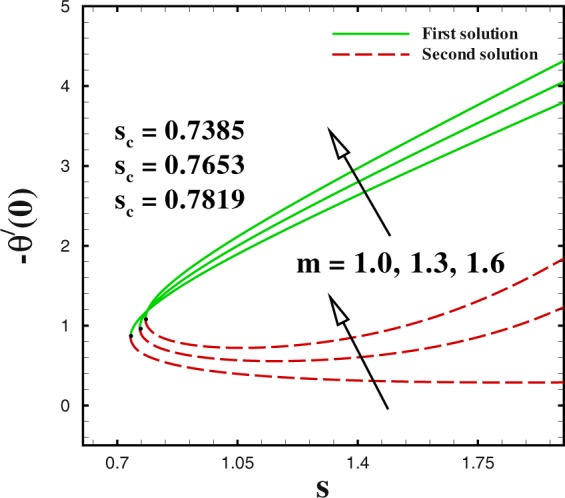
Impact of *m* on Nusselt number.

**Figure 7:**
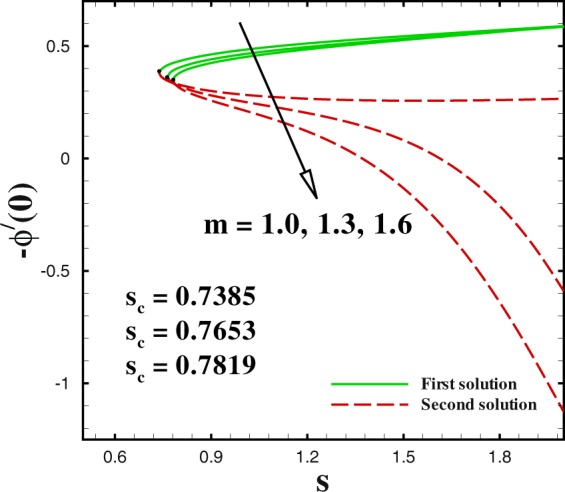
Impact of *m* on Sherwood number.

**Figure 8:**
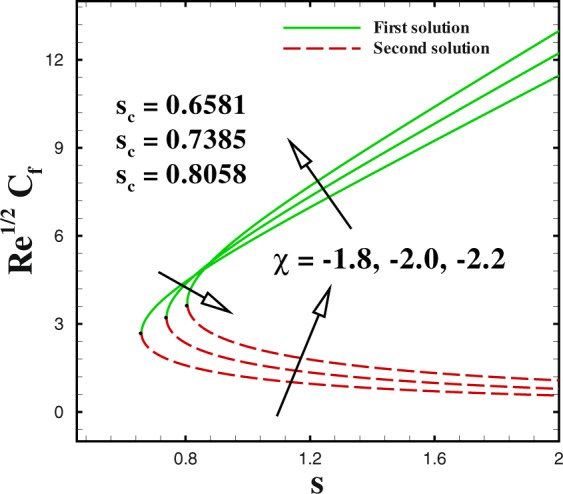
Impact of *χ* on skin friction.

**Figure 9:**
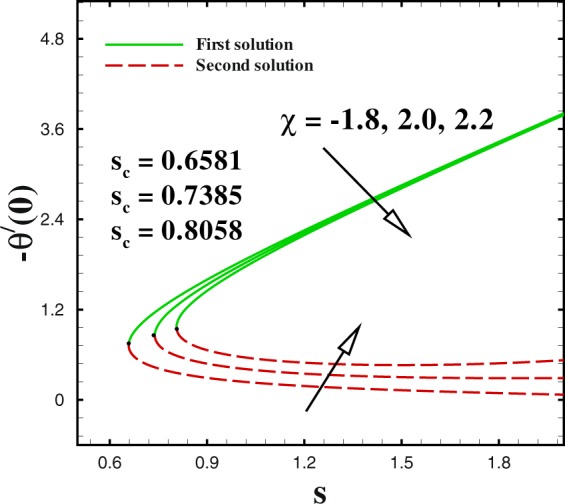
Impact of *χ* on Nusselt number.

**Figure 10:**
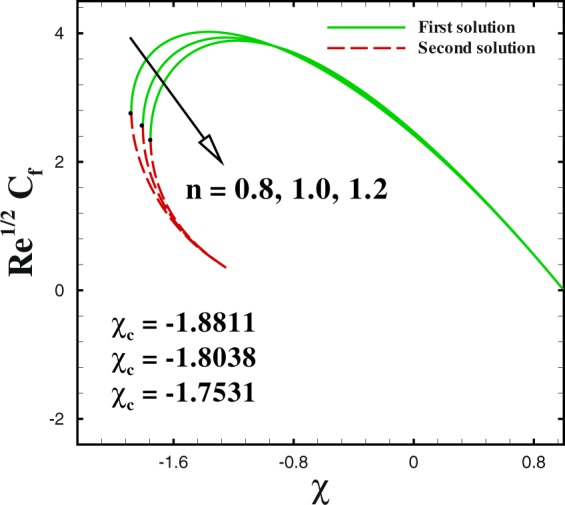
Impact of *n* on skin friction.

**Figure 11:**
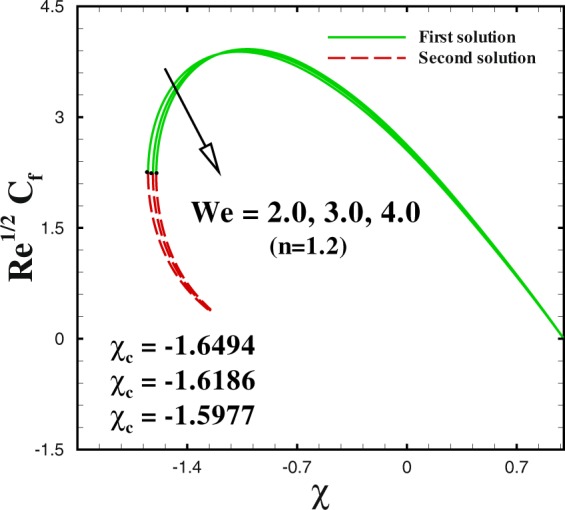
Impact of *We* on skin friction.

**Figure 12:**
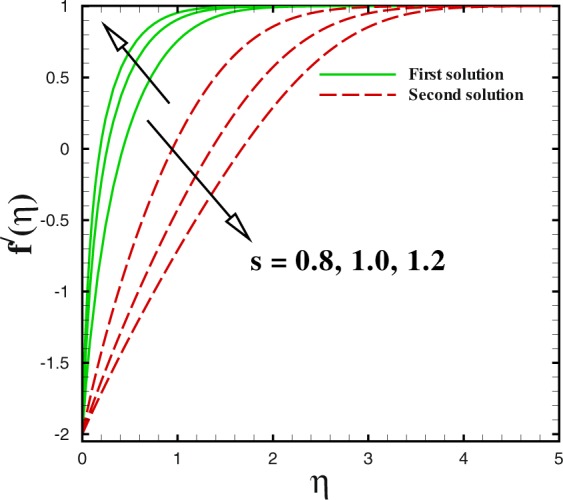
Dual velocity profiles for different values of *s*.

**Figure 13:**
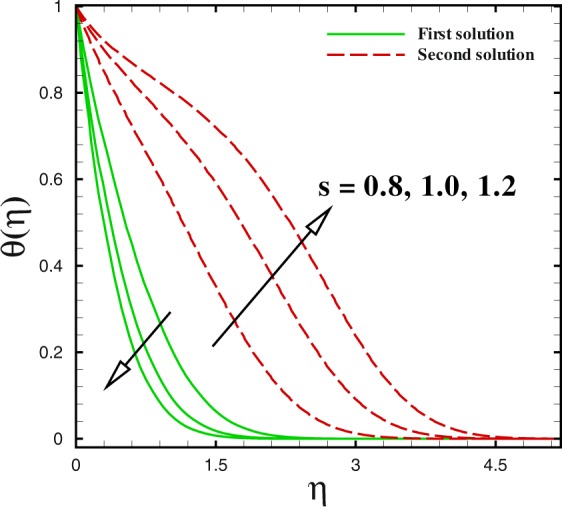
Dual temperature profiles for different values of *s*.

**Figure 14:**
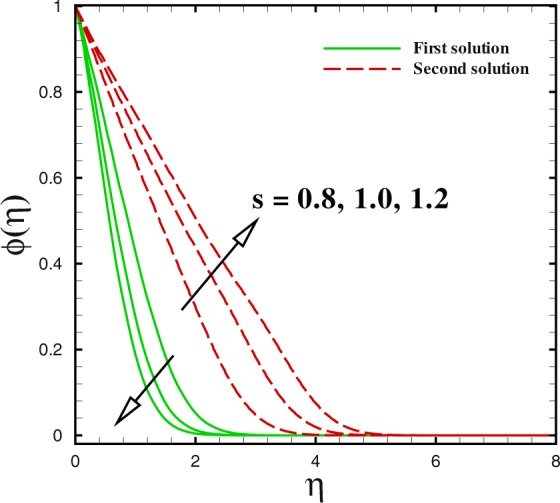
Dual concentration profiles for different values of *s*.

**Figure 15:**
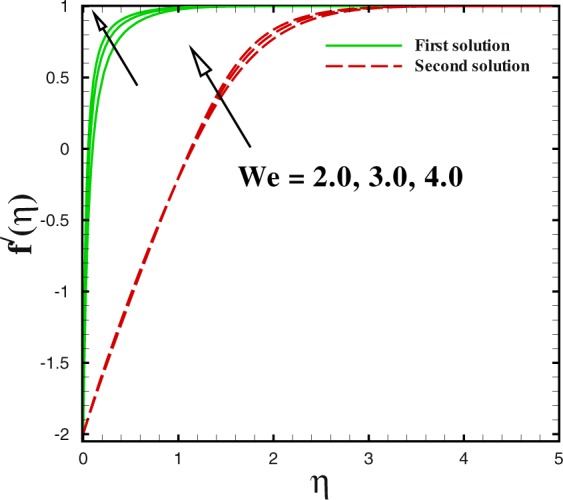
Dual velocity profiles for different values of *We*.

**Figure 16:**
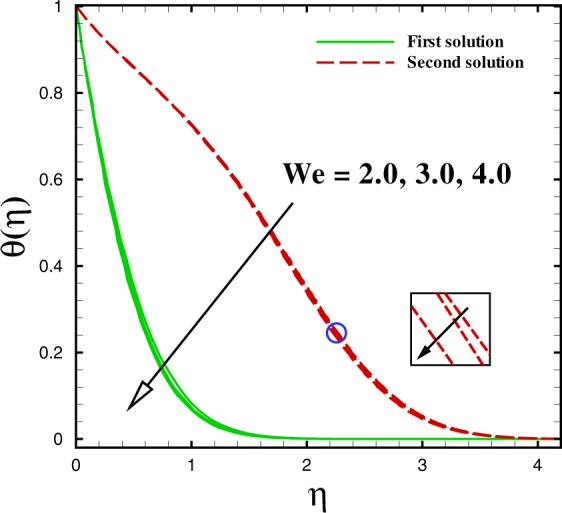
Dual temperature profiles for different values of *We*.

**Figure 17:**
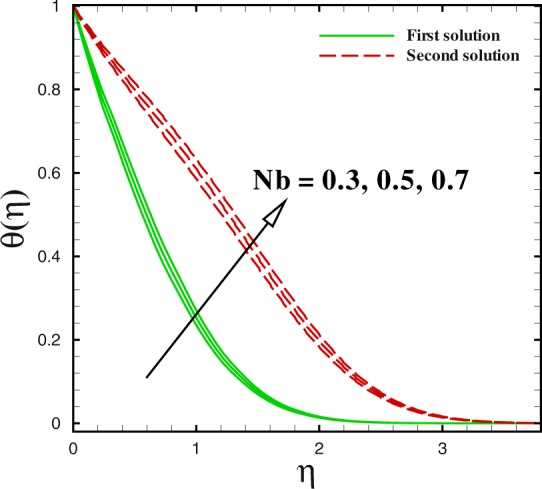
Dual temperature profiles for different values of *N*_*b*_.

**Figure 18:**
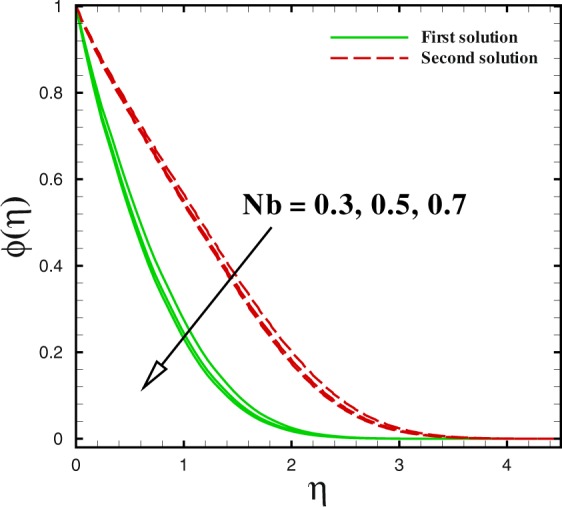
Dual concentration profiles for different values of *N*_*b*_.

**Figure 19:**
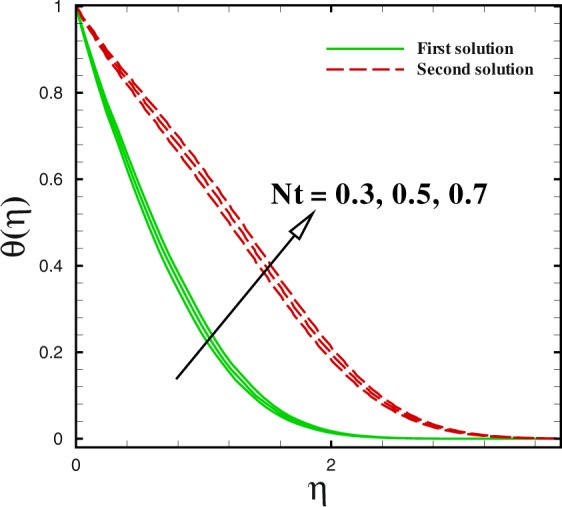
Dual temperature profiles for different values of *N*_*t*_.

**Figure 20:**
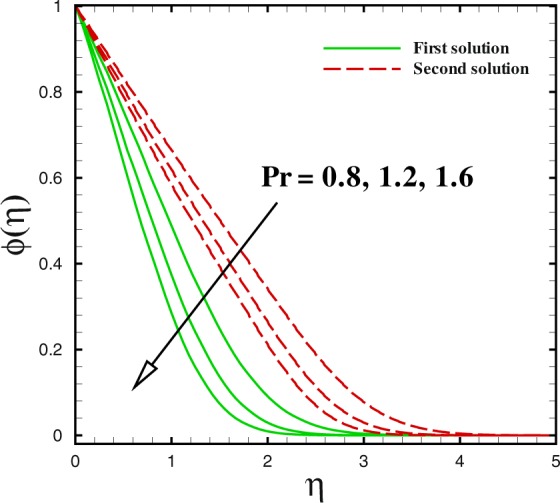
Dual concentration profile for different values of Pr.

In the accompanying segment, our fundamental objective is to comprehend the physics of the numerical model through tables and graphic structures. To dissect the effects the different flow parameters likewise the Weissenberg number *We*, the suction parameter *s*, the stretching/shrinking parameter *χ*, Prandtl number Pr, the Lewis number *Le*, the Brownian motion *Nb* and the thermophoresis parameters *Nt* on skin friction coefficient, local Nusselt number and local Sherwood number. Moreover, we apportioned sensible numerical values to the supervising parameters with a true objective to get a comprehension into the velocity, temperature and concentration profiles. We set default values of supervising parameters as *We* = 0.5, *m* = 1.0, *n* = 0.5, *χ* = −2, *s* = 0.8, Pr = 1.0 *Nb* = 0.1, *Nt* = 0.1, *Le* = 1.0. The skin friction coefficient ${{\rm{R}}{\rm{e}}}^{1/2}{C}_{f},$ Nusselt number −*θ*′(0), Sherwood number −*ϕ*′(0), velocity *f*′(*η*), temperature *θ*(*η*) and concentration *ϕ*(*η*) profiles are given through Figs 2–20. Solid line stands for the first solution and dash line for the second solution.

### Physically concerned quantities

The impact of sundry supervising flow parameters on skin friction coefficient, the rate of heat transfer and the local Sherwood number are respectively discussed through several graphs. The focus of this dissection is to captured dual solution. solid line represents for the First solution (upper branch solution) and dash lines for the second (lower branch solution). Dual solution exists in some cases. One of them is that when flow flows over a moving surface. As expressed by a few authors for instance by Lio^23^, Fang^24^ and Khan *et al*.^28^ who explicated the nature of dual solution occurrence. Furthermore, it uncovers a fascinating actuality that both branches solution arrive at an end at a specific value of suction parameter *s* and stretching/shrinking parameter *χ* are known as critical value (critical point) (*s*_*c*_) and (*χ*_*c*_) respectively. At that critical point only one solution can be found for both branches. It should be realized that dual solution exists in the range *s* ≤ *s*_*c*_ and *χ* ≤ *χ*_*c*_ in the beyond of critical value i.e., *s* < *s*_*c*_ and *χ* < *χ*_*c*_, no solution exists. Many researchers^33–36^ performed the stability solution for the both branches. It may be concluded that first branch of the solution is stable and physically reliable while the lower branch is unstable and not physically meaningful.

Figures 2 to 4 shows the impact of Weissenberg number *We* on skin friction coefficient ${{\rm{R}}{\rm{e}}}^{1/2}{C}_{f}$ Nusselt number −*θ*′(0) and Sherwood number −*ϕ*′(0) by keeping the other parameters fixed. It is seen that dual solution exists in all the graphs and both solutions are separated by a critical value. For increasing values of Weissenberg number *We*, the skin friction coefficient increases in upper branch solution and a converse pattern is noted in the lower branch solution, as seen in Fig. 2. The critical values reduces with respect to mass suction parameter with an enlargement in Weissenberg number *We*. It can be visually perceived that dual solution exists in the range *s*_*c*_(=−4.162, −4.386, −4.4425) ≤ *s* when *We* = 2.0, 3.0, 4.0. Figure 3 shows that a similar behaviour is observed for local Nusselt number for varying values of Weissenberg number *We*. The dual profiles for local Sherwood number −*ϕ*′(0) are depicted in Fig. 4. An enhancement is noted in upper branch solution for higher values of Weissenberg number. While, in case of lower branch solution, the rate of mass transfer −*ϕ*′(0) reduces with increasing values of *We*. It is important to highlight that the critical values remain unchanged for the friction, heat and mass transfer coefficients for similar values of mass transfer parameter *s*.

The profiles of skin friction coefficient, local Nusselt number and local Sherwood number for varying values of *m* are plotted in Figs 5 to 7 against the suction parameter *s*. These Figs render that the dual solution for skin friction coefficient, local Nusselt number and local Sherwood number are significantly influenced by *m*. These Figs tell us that dual nature of solutions occurs for fixed values of m i.e., *m* = 1.0, 1.3, 1.6. The critical value of mass transfer parameter *s*_*c*_ varies from 0.7385 to 0.7819, as *m* changes from 1.0 to 1.6. We clarify that the both (first and second) solutions of both skin friction and local Nusselt number increases by higher *m*. To discuss the influence of *m* on local Sherwood number −*ϕ*′(0), we plotted Fig. 5 for different values of *m*(=1.0, 1.3 and 1.6) with regard to the suction parametric *s*. We observe that increasing values of *m* causes a reduction in local Sherwood number for both upper and lower branch solutions. It is seen that no solution exists in the range *s* < *s*_*s*_.

We sketched Figs 8 and 9 to illustrate the effect of shrinking parameter *χ* on skin friction coefficient and local Nusselt number. As expected, multiple solution exists in the range *s*_*c*_(=0.6581, 0.7385, 0.8058) ≤ *s* when *χ* = −1.8, −2.0 and −2.2. In Fig. 8 both solutions give a reducing behaviour for skin friction when shrinking parameter *χ* changes from −1.8 to −2.2. The impact of shrinking parameter *χ* on −*θ*′(0) is seen in Fig. 9. It can be derived that the local Nusselt number decreases with an increment in *χ* for upper branch solution. While, in case of lower branch solution, Nusselt number increases with higher *χ*. To analyse the skin friction at the control surface for different values of *n* parameter and Weissenberg number *We* of the shrinking case (*χ* = −2.0), results are sketched in Figs 10 and 11. The behaviour of skin friction ${{\rm{R}}{\rm{e}}}^{1/2}{C}_{f}$ boosted down with the increment of *n*(=0.8, 1.0, 1.2) and *We*(=2.0, 3.0, 4.0) respectively for the first solution. Additionally, the greater rate of *n* and *We* parameters give the decreasing rate of critical point as shown in these Figs.

### Boundary layer flow profiles

Figures 12 to 14 unveil the influence of mass suction parameter *s* on velocity *f*′(*η*), temperature *θ*(*η*) and concentration *ϕ*(*η*) distributions, respectively using other parameters fixed. For the case of flow over shrinking surface, the velocity profile rises with the increment of suction parameter *s* in the first branch of solution. For lower branch, it is diminishing. Moreover, the momentum boundary layer thickness reduces in the upper branch and enhances for the lower branch solution (see Fig. 12). Figure 13 is pictured to see the impact of mass transfer parameter *s* on temperature distribution *θ*(*η*). It gives reduction behaviour in the first solution with increase in *s* while a quite different behaviour can be viewed for the second solution. Additionally, larger rate of suction causes to be reduced the thickness of momentum boundary in the first solution. But it increases with *s* for the second solution. The concentration of nanoparticle *ϕ*(*η*) gives a lesser behaviour in the first branch of solution with *s* = 3.0, 4.0 and 5.0. It can be observed that for second branch of solution, concentration boundary layer thickness enlarges with suction *s*.

The effect of Weissenberg number *We* on velocity and temperature profiles are drawn in Figs 15 and 16. With the increment in Weissenberg number *We*, the fluid velocity boosts up in both solutions which is expounded in Fig. 15. The temperature profile *θ*(*η*) is sketched to see the impact of *We*. Both solutions are depressed with higher *We*. In both sketches, it is concluded that both the momentum and thermal boundary layer thickness reduces to the lagging value of Weissenberg number (*We* = 2.0, 3.0, 4.0).

Figures 17 to 19 are sketched to watch the variance in temperature and concentration profiles against *η* under the action of nanofluids parameters. From Fig. 17, the temperature improves for large value of Brownian motion parameter *Nb* in both solutions. Additionally, at this encroachment, the thermal boundary layer thickness enhances. A quite different trend can be noted for concentration profile in Fig. 18. The thermophoretic effect can be discern in Fig. 19. With the development in thermophoresis *Nt* shows the increment in fluid temperature *θ*(*η*) and the thermal boundary layer thickness elevates with advancement of *Nt* in both solutions. The influence of Prandtl number Pr on temperature profiles is described through Fig. 20. Dual solution occurs with respect to *η*. With the advancement in Pr, the concentration of nanoparticle declines in both solution in the case of shrinking surface (*χ* = −2.0). Also, it can be conducted that thickness of concentration boundary layer diminishes in both solution.

## Main Findings

This study work presents a numerical-based survey for the subsistence of dual homogeneous attribute solutions for flow on a moving crustal surface in nanofluids. Two-dimensional axisymmetric flow of Carreau fluid model is utilized and bvp4c function in MATLAB is used to gain with effort the dual solutions. In the end, there are some paramount outcomes for this discourse got as:

1. Dual solution exists in the case of moving surface.
2. At the sheet, the local Nusselt number and local Sherwood number is, respectively greater for higher values of Weissenberg number in the upper solution while decline in the lower solution.
3. The skin friction coefficient is diminishing deportment for the escalating value of stretching parametric quantity.
4. The parameter foresee the escalating impact in the upper branch solution.
5. It is uncovering that heightening value of Weissenberg number foresee the decline of momentum and thermal boundary layer thickness.
